# Medico-Legal Insanity

**Published:** 1857-07

**Authors:** 


*Medico-Legal Insanity.— Trial of James Getty.*—James Getty, indicted for the murder of Daniel Albert, was tried in the Court of Oyer and Terminer of Erie County, on the 24th of May and succeeding days, before Justice Bowen. Mr. District Attorney Humphrey and Hon. Solomon G. Haven appeared for the people; Mr. Albert Sawin, Eli Cook and J. G. Hoyt, Esqrs., for the defence.

It was proven by the prosecution that on the 13th of March, at about noon, Daniel Albert was driving along the public road, when Getty approached him with a gun. When about twice the length of the gun distant, Getty shot Albert in the thigh, and cocked the other barrel. Albert jumped off his wagon, seized the gun and tried to get it away or hold the muzzle from him; Getty drew back, thus bringing the gun in range, and fired, the shot took effect in the other thigh, and at the same time blew off his thumb. Getty said "there!" the first words spoken. Albert fell on the snow, Getty brandishing the gun over him. Albert again seized the gun: Getty told him to let go, pulled it from him and walked away. Getty then went into the house, put down the gun, and the murder being mentioned his wife asked him if it was possible he had done such a thing: he replied that he was sorry for it. Some one came and asked him the news; he replied, "The strangest news out here." He had his supper, and was arrested directly afterward.

It was also proven that an old enmity had existed between the prisoner and deceased, and that on many occasions Getty had manifested much bitterness toward Albert.

The defence was insanity. Several witnesses testified to the general good and amiable character of Getty, and that he had never had difficulty with any one except Albert.

Levant Getty, a son of the prisoner, testified that after the death of his sister in August, 1854, his father had been very solemn; in June, 1855, he took a pistol and went out to the barn, came back in half or three-quarters of an hour, looking very strange, and sat up till one in the morning without saying a word; he acted moody after this, and wished to be alone; in 1855 witness saw him talking to himself in the barn, looking excited and pale; in May or June, 1856, he acted strange, and during last summer he would spend much time wandering in the woods or lots; was careless of his business and taciturn; complained of his head. In the fall witness found him sobbing in the barn; he was off for four days once without cause. Once found him watching the house with a gun, at 12 or 1 o'clock in the morning; the family watched him continually; he could not add up figures; once, without cause, he advertised in the *Patriot and Journal*, to warn people not to trust his family. There had been no trouble to cause this.

Manuel Henshaw testified to irregular conduct and insane appearance of the prisoner.

George W. Gilman says: In the spring of 1856, he was much excited at seeing Albert across the way. He said "By G—d, I am a white man and live in a free country." His wife soothed him, and got him in the house. After that he went to the barn and shut himself in; came back and ate his supper, but did not speak again that evening. Last February Getty came to witness and asked him why he reported that he (Getty) had taken a rope and gone to the barn to hang himself. Witness denied it, but told him that he believed he was an insane man, and would swear to it. A conversation ensued, in which Getty denied recollection of circumstances mentioned by witness. Witness told him that he had advised his family to send him to an insane asylum. Getty became angry, and accused witness of conspiracy with Albert.

The mother of the prisoner testified to a conversation with her son about his suspicions of his wife. She told him his troubles were imaginary, but at his solicitation sounded the neighbors, and learned nothing. His appearance was changed, and he became melancholy. Testified to finding him feeling bad in the barn at the time when he shut himself up; he said he could not live so.

He got up nights sometimes, said he could not sleep, complained of his head. At another time he said he was going out of the country for five years. Several times he wept in talking of his troubles. Getty brought witness into Buffalo to take the cars for Batavia. Unexpectedly to her, he went with her, saying he never should go back to Hamburgh; that he had met Albert, had had words from him, and would have shot him if he had had a pistol. He wept on the cars. Last summer there was talk of sending him to the asylum. He is 53 years old.

Other evidence of a similar character was produced. It was proven that he was subject to sudden and causeless bursts of tears; that he guarded the house at night with a gun; that he had causeless suspicions of the fidelity of his wife; was gloomy, suspicious and morose in his manner, that he had several times declared a purpose to leave the country, and on a visit to the west had alarmed his relatives by insane deportment. The evidence of Mr. Perry G. Parker is important. Mr. Parker testified to a series of interviews with Getty last fall as his legal adviser. Getty wished to make a will cutting off his wife's dower; said that there was a conspiracy against him, and that he must leave the country and the world. He wanted to go west, and said he should be killed on the cars. Witness then told him he might go by steamboat; Getty said that would make no difference. He wept and seemed sad and gloomy. In a subsequent interview he complained of a political conspiracy, that a meeting had been held at the *Republic* office, at which most of the city and county officials were present, and letters were read concerning him. He wished witness to discover why some officers were not invited; said a policeman who had been discharged on his account had something to do with it.

These were the facts in evidence on which the medical witnesses were called to base their opinion.

Prof. James P. White testified that he had known the prisoner more or less from a boy; had visited him in jail twice with Dr. Flint. His recollections of early life were correct. When questioned he would answer f.eely as to his quarrels with Albert and the act of killing him, but the dominant idea in his mind was his present happy frame of mind, on account of a great religious change he had experienced in jail: he constantly reverted to this subject; he admitted that he had no cause which he could tell for suspicion of his wife, but could not resist the conviction. He had never seen Albert about his house, but knew he had been. He took no interest in the murder, left that all with God; did not know whether it was right or not, but he was happy now in his religious feelings. At the request of Dr. Flint he explained that he could not sleep the first night in jail, but spent the night in prayer; and so, too, the second night; when, though the cell was dark, he saw an eye, much larger than a human eye, which he knew to be the eye of God shining in the darkness. He felt as if his feet were put in very hot water, chills ran all over him, and his head was relieved; he became crystalized, and then went to sleep. He said that he was not insane now, and did not admit or deny that he ever had been. He knew that his defence was on the ground of insanity. Dr. White then testified, as an expert to his opinion that Getty was now, and has been for a year past, of unsound mind. He gave a *resumé* of his reasons for that opinion in a very clear and intelligible manner.

(We regret that our report of Dr. White's testimony is so meagre. We are compelled to do even greater injustice to Dr. Flint.)

Prof. Austin Flint sworn. Witness has read a statement furnished him by the counsel for prisoner and subsequently visited Getty in jail. He believes that Getty has been insane for a year past and is now insane.

Dr. Flint summed up the symptoms on which his opinion was founded; the melancholy, change of habits, religious exaltation, fear and suspicion of friends, and his physical appearances. He had carefully examined the symptoms of Getty's case, keeping the thought of simulated insanity in view; he saw no evidence of deception in him.

*Cross-examined.*—A person insane on any one point cannot be considered as entirely sane on any point. Monomania is a symptom of diseased mind.

The next witness called was Dr. George Cook, the accomplished physician of Brigham Hall, an excellent private insane asylum at Canandaigua. Witness was educated as a physician, was associated with Dr. Brigham at the Utica Asylum, one and a-half years. Is now in charge of a private lunatic asylum at Canandaigua; has given his entire attention to insanity for nine years. Insanity means a chronic nervous disease which changes the natural feeling, thinking or acting, and impairs or destroys the free will of the patient; it renders him incapable of fully directing and controlling his actions, and of correctly appreciating his responsibility to himself, his family, friends, society or the laws; enumerated the special symptoms in Getty's case, bearing on the question of insanity, at considerable length, reciting from the evidence given, from his examination of the patient, and his conduct during the trial; from all this he said: "All these conversations, as well as other facts in the case, are symptoms which to my mind converge to one common centre, which is the existence in prisoner's mind of delusions of fear and suspicion. I have no hesitation in expressing my opinion that the prisoner is insane. I say this without any doubt. If Getty's case had been presented to me months before his arrest, I should have had no hesitation in saying that he was not only insane, but dangerously so at any time after his visit to the west."

*Cross-examined.*—Mr. Haven examined the witness at considerable length as to the existence of monomania leaving the mind sound on all points except the particular delusion.

Dr. Cook testified to the same purport as Dr. Flint, that a monomania necessarily involved some degree of unsoundness of the reasoning powers; he said that he would not apply the term "uncontrollable impulse" to any condition of Getty's at any one time, but he thought that at several times the delusion of fear upon him was so strong that he acted entirely under the influence of that fear, just as a sane man might act, not uncontrollably but under a strong conviction.

In reply to a question, asked for the satisfaction of public feeling in Hamburgh, witness stated that any person visiting a lunatic asylum might meet patients and converse with them occasionally for months, and never detect a symptom of insanity, and yet the patient be a dangerous lunatic all the time.

In reply to another question, he stated his opinion that some degree of material change in the brain or its membranes, always accompanied insanity, but it might be very slight, a mere difference in shade of color, or even not perceptible.

The case here closed on the part of the defence.

The counsel waived the privilege of summing up the case. The charge of the judge was favorable to the plea of insanity, and the jury returned a verdict of "not guilty by reason of insanity," after an absence of only five minutes.

The history of this melancholy case is another evidence that a dangerous degree of insanity may exist for a long period of time, without being manifest to those not intimately associated with the patient. The neighbors of Mr. Getty were almost unanimous in the belief that his insanity was feigned. They had done business with him in the ordinary avocations of life, without discovering any sign of insanity; and it was only the members of his immediate family, or those to whom he confided his feelings confidentially, that had any suspicion of his mental soundness.

An unfortunate circumstance led the family to conceal Mr. Getty's insanity. It at first took the form of suspicion of the fidelity of his wife, an estimable woman, and she, unwilling to lift the veil from her family sorrows, would not consent to his removal to an asylum last fall. This seemed at the time the proper course, but it should never have been adopted without the consent of an intelligent physician, and his assurance that his insanity was harmless. This is doubtless a source of poignant regret to the relatives now.

The clear, decided testimony of the medical witnesses is worthy of mention. Their evidence was given in the face of popular opinion, which had become incensed by recent abuses of the plea of insanity. Fortunately for the ends of justice, Mr. Sawin opened the defence by placing it distinctly on the ground that no irritability or violence of temper, no eccentricity, and no brooding over injury should be interpreted as insanity. Neither did they believe in, or claim the presence of a moral insanity, driving the prisoner to the commission of crime.

On the return of the jury the prisoner received the verdict with that entire absence of interest or emotion which had marked his conduct throughout. A writ of *de lunatico inquirendo* was moved, but subsequently set aside, and the prisoner was ordered to be taken to the State Asylum at Utica, for medical treatment.

